# A multi-source approach to determine SMA incidence and research ready population

**DOI:** 10.1007/s00415-017-8549-1

**Published:** 2017-06-20

**Authors:** Ingrid E. C. Verhaart, Agata Robertson, Rebecca Leary, Grace McMacken, Kirsten König, Janbernd Kirschner, Cynthia C. Jones, Suzanne F. Cook, Hanns Lochmüller

**Affiliations:** 1 John Walton Muscular Dystrophy Research Centre, Institute of Genetic Medicine, Newcastle University, Central Parkway, Newcastle upon Tyne, NE1 3BZ UK; 2 Clinical Trials Unit, Medical Center, University of Freiburg, Freiburg, Germany; 3 Department of Neuropediatrics and Muscle Disorders, Medical Center, University of Freiburg, Freiburg, Germany; 4 Biogen MA Inc., Cambridge, USA; 5 Epidemiology Associates LLC, Chapel Hill, USA

**Keywords:** Spinal muscular atrophy, Incidence, Prevalence, Genetic laboratories, Registries

## Abstract

In spinal muscular atrophy (SMA), degeneration of motor neurons causes progressive muscular weakness, which is caused by homozygous deletion of the *SMN1* gene. Available epidemiological data on SMA are scarce, often outdated, and limited to relatively small regions or populations. Combining data from different sources including genetic laboratories and patient registries may provide better insight of the disease epidemiology. To investigate the incidence of genetically confirmed SMA, and the number of patients who are able and approachable to participate in new clinical trials and observational research, we used both genetic laboratories, the TREAT-NMD Global SMA Patient Registry and the Care and Trial Sites Registry (CTSR). In Europe, 4653 patients were genetically diagnosed by the genetic laboratories in the 5-year period 2011 to 2015, with 992 diagnosed in 2015 alone. The data provide an estimated incidence of SMA in Europe of 1 in 3900–16,000 live births. Patient numbers in the national patient registries and CTSR were considerably lower. By far, most patients registered in the national patient registries and the CTSR live in Europe and are reported to have SMA type II. Considerable differences between countries in patient participation in the registries were observed. Our findings indicate that not all patients with SMA are accessed by specialist healthcare services and these patients may not have access to research opportunities and optimal care.

**Electronic supplementary material:**

The online version of this article (doi:10.1007/s00415-017-8549-1) contains supplementary material, which is available to authorized users.

## Introduction

Spinal muscular atrophy (SMA) is an autosomal recessive neuromuscular disorder. In SMA, a mutation in the survival motor neuron gene (*SMN1*) at locus 5q13.2 leads to degeneration of alpha motor neurons, resulting in progressive muscular weakness [1]. The majority of patients (92%) have a homozygous *SMN1* deletion. In the remaining patients, point mutations are found or SMA is caused by mutations in other genes [2]. A homologous copy of the *SMN1* gene, the *SMN2* gene, is presented at the same chromosome, which is capable of producing about 10–20% of full-length SMN protein [3, 4]. *SMN2* is presented in varying copying copies, which plays a role in the heterozygosity of the phenotype [5–7].

The clinical classification system is based on the age of symptom onset and the maximum motor function achieved [8, 9]. Type I SMA (Werdnig–Hoffmann disease) has an onset in the first months of life. Patients are never able to sit without support and without ventilatory support most patients will not survive after 2 years [10, 11]. Type II patients, with onset between six and 18 months of age, reach the ability to sit independently. Type III (Kugelberg–Welander disease) is less severe, with onset after 18 months of age. Patients gain the ability to walk independently and usually survive into adulthood [8, 9, 12].

According to the literature, SMA due to *SMN1* mutations has an incidence of approximately 1 in 10,000 newborns [13–18] and a prevalence of approximately 1–2 per 100,000 persons [13, 19]. Most patients suffer from SMA type I [15]. However, no worldwide studies have been performed. Numbers are mainly based on small studies, many of which predate genetic testing and with classification schemes that have changed over the years, highlighting the need for contemporary data.

This study aimed to estimate the worldwide incidence of SMA and the research ready and accessible population, using a by combination of multiple sources, including genetic laboratories and patient and clinical registries.

## Materials and methods

### Genetic laboratories

Genetic laboratories testing for *SMN1* were identified using publically available information as well as expert input and validation using the following sources: the Eurogentest/Orphanet database of diagnostic laboratories, the European Directory of DNA Diagnostic Laboratories (EDDNAL), the laboratory database via GeneTests.org, the Genetic Testing Registry (GTR) from NCBI, several country-specific websites, and personal communication with patient registry curators and researchers from specific countries.

Responses from genetic laboratories were collected via an online survey (http://www.surveymonkey.net) to determine the number of patients with a genetically confirmed diagnosis. The structured survey included questions about diagnostic techniques, total numbers of positive diagnoses, excluding prenatal, in 2015 and in the 5-year period (1 January 2011–31 December 2015). The survey was distributed via personalised emails. Two reminders were sent out and up to three further follow-ups were performed fortnightly via telephone and email. In relevant countries, local experts were consulted to determine the important genetic laboratories and their sizes.

### TREAT-NMD Global SMA Patient Registry and Care and Trial Site Registry

The TREAT-NMD Alliance (http://www.treat-nmd.eu) is an international network for rare inherited neuromuscular disorders providing an infrastructure to increase international collaboration between clinical and scientific experts, accelerate therapy development, improve patient care with best-practice consensus guidelines, and deliver services for industry [20]. Two key elements of TREAT-NMD are the Global Patient Registries and the Care and Trial Site Registry (CTSR).

The Global SMA Patient Registry consists of national patient registries, collecting a number of mandatory and highly encouraged items (genetic and clinical) of genetically confirmed patients. These can be self-reported and/or provided by professionals. More than 5000 SMA patients worldwide have been enrolled in TREAT-NMD-associated registries [21]. The TREAT-NMD Global Database Oversight Committee (TGDOC), comprised of representatives of national registries governs the Global SMA Patient Registry. The TGDOC reviews all enquiries to the Global Patient Registry and approved the enquiry of this study.

The CTSR is an online database of NMD‐specialist clinical sites and medical centres, providing information about the facilities, equipment, personnel, and experience of these sites as well as about patient cohorts [22]. Currently, more than 330 expert centres regularly provide updates to the CTSR (personal communication).

We requested information about living patients from the Global Patient Registry and the CTSR to determine the accessible SMA population. An enquiry was submitted to the Global Patient Registry for the total number of genetically diagnosed patients alive on 1 September 2015, stratified by type of SMA (I–III), current age, and sex. The CTSR provided data on the number of clinically diagnosed patients per site on 15 December 2015, stratified by SMA type (I–III) and age. There is known overlap between these two registries.

### Data analysis

For consistency purposes, population data for all countries included in the analysis were extracted from the United Nations [23], which report population numbers per year (as of 1st July) and the number of live births in periods of 5 years (i.e., 2011–2015). To estimate the number of live births for 2015, the number of live births for the period 2011–2015 was divided by five. This approximation was used, because not every country has a national statistical office providing accurate data per year. We calculated incidence (the proportion of newborns who have confirmed SMA; the measure estimated herein is not a true incidence or incidence rate, but rather the prevalence at birth of SMA. Nevertheless, as much of the SMA literature uses the nomenclature of ‘incidence’, we use it here) by dividing the number of positive tests by the number of live births in the same period and prevalence by dividing the number of patients at the measured timepoint by the total population. Confidence intervals were calculated based on the Poisson distribution.

## Results

### Incidence in Europe

Initially, the survey was distributed to genetic labs worldwide; however, due to low level of response and difficulties with identifying all laboratories in the countries outside of Europe, it was decided to focus on Europe. Here, we present the results of the survey responses received from 122 laboratories across 27 countries. In total, 4653 patients were genetically diagnosed with SMA in the 5-year period 2011–2015, of which 992 in 2015 alone.

Sufficient information (response from laboratories responsible for >80% of all SMA tests, presumably yielding more complete data on genetically confirmed SMA patients) was obtained from 18 countries. In these countries, there were 22.3 million live births in the period 2011–2015, of which 4.5 million were in 2015. In 2015, 784 new SMA cases were identified and 3776 over the period 2011–2015 (for one country, only patient numbers for 2015 were available). Incidence rates were comparable in 2015 and 2011–2015 (Table 1). The median incidence of SMA in the period 2011–2015 was 11.9 per 100,000 [range 6.3–26.7 per 100,000 (~1 in 3900–16,000)].

**Table 1:** Incidence rate from genetic laboratories

Country	Part of Europe	No. of responses	2015				2011–2015^a^			
			No. of patients	No. of live births	Incidence (per 10^5^)	95% CI	No. of patients	No. of live births	Incidence (per 10^5^)	95% CI
Finland	Northern Europe	1/1	6	57,949	10.4	8.9–11.9	30	289,746	10.4	7.0–14.8
Denmark	Northern Europe	2/2	7	58,528	12.0	4.8–24.6	29	292,640	9.9	8.6–14.3
United Kingdom	Northern Europe	11/12	88	804,083	10.9	8.8–13.5	438	4,020,416	10.9	9.9–12.0
Ireland	Northern Europe	1/1	4	71,787	5.6	1.5–14.3	24	358,933	6.7	4.3–10.0
The Netherlands	Western Europe	2/2	17	177,029	9.6	5.6–15.4	89	885,145	10.1	8.1–12.4
Belgium	Western Europe	6/8	17	128,767	13.2	7.7–21.1	73	643,834	11.3	8.9–14.3
France	Western Europe	10/12	148	787,151	18.8	15.9–22.1	816	3,935,757	20.7	19.3–22.2
Germany	Western Europe	26/30	186	671,455	27.7	23.9–32.0	857	3,357,275	25.5	23.8–27.3
Italy	Southern Europe	19/30	115	513,149	22.4	18.5–26.9	550	2,565,747	21.4	19.7–23.3
Slovenia	Southern Europe	1/1	3	21,577	13.9	2.9–40.6	17	107,884	15.8	9.2–25.2
Croatia	Southern Europe	1/1	8	41,770	19.2	8.3–37.7	44	208,850	21.1	15.3–28.2
Bulgaria	Eastern Europe	1/2	17	68,692	24.7	14.4–39.6	77	343,462	22.4	17.7–28.0
Hungary^b^	Eastern Europe	1/1	21	92,761	22.6	14.0–34.6	–	–	–	–
Slovakia	Eastern Europe	2/3	10	56,893	17.6	8.4–32.3	45	284,463	15.8	11.5–21.2
Czech Republic	Eastern Europe	5/6	11	107,689	10.2	5.1–18.3	64	538,446	11.9	9.2–15.2
Poland	Eastern Europe	6/9	54	401,133	13.5	10.1–17.6	240	2,005,665	12.0	10.5–13.6
Ukraine	Eastern Europe	2/2	52	487,875	10.7	8.0–14.0	240	2,439,376	9.8	8.6–11.2
Greek-Cyprus	Europe/Western Asia	1/1	0	9200^c^	0.0	0.0–40.1	3	47,582^c^	6.3	1.3–18.4

Countries ordered by geographical location

^a^In case a laboratory could not provide data for 2011–2015, the number for 2015 was multiplied by five as an estimation (unless laboratory indicated, they started testing at a later timepoint)

^b^No numbers are given for 2011–2015, since the only laboratory in Hungary stated that they started testing in 2015

^c^For Cyprus, population data were used from the Statistical Service of Cyprus (CYSTAT) instead of the United Nations to obtain numbers for the Greek part of Cyprus only

### Prevalent cases ready for participation worldwide

**Fig. 1:**
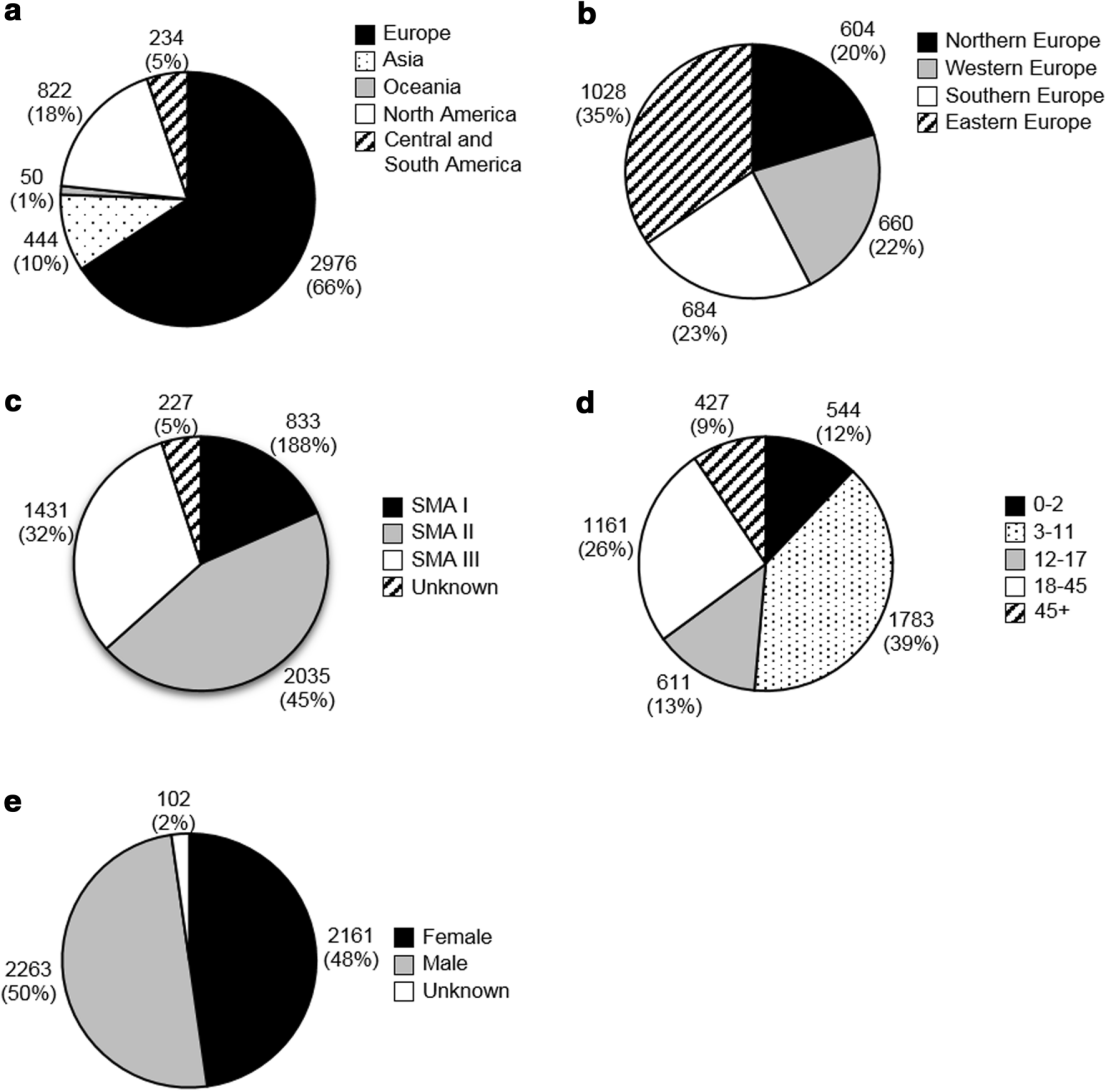
Patients in the Global SMA Patient Registry. Number of patients and percentage of total is indicated next to each part. Origin of patients worldwide (**a**) and subdivision in Europe (**b**). Europe: Northern Europe: Denmark, Finland, Ireland, Lithuania, Norway and United Kingdom. Western Europe: Austria, Germany, the Netherlands and Switzerland. Southern Europe: Italy, Serbia and Spain. Eastern Europe: Bulgaria, Czech Republic, Hungary, Poland, Russian Federation, Slovakia and Ukraine. Asia: China and Turkey. Oceania: Australia and New Zealand. North America: Canada and the United States. Central and South America: Argentina, Brazil and Mexico [23]. **c** SMA type. **d** Age group. For comparison age groups were chosen to match CTSR data. **e** Gender

The enquiry into the Global SMA Patient Registry provided data from 26 national registries, representing 29 countries (some registries cover more than one country) worldwide. The registries that responded contained a total of 4526 genetically confirmed patients. The results by region, SMA type (I–III), age, and gender are summarized in Fig. 1.

**Fig. 2:**
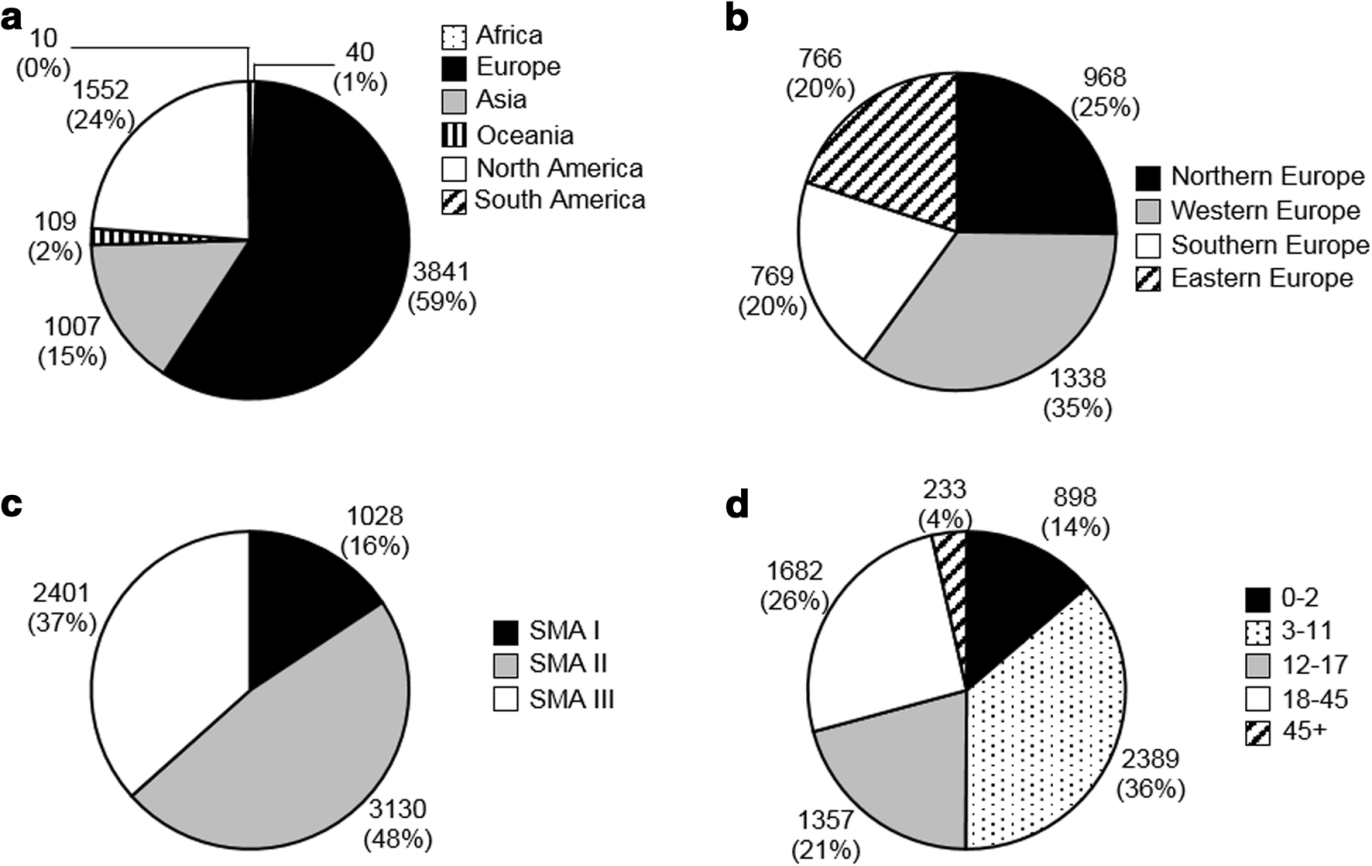
Patients in the Care and Trial Site Registry. Number of patients and percentage of total is indicated next to each part. Origin of patients per continent (**a**) and subdivision in Europe (**b**). Europe: Northern Europe: Denmark, Finland, Ireland, Norway, Sweden and United Kingdom. Western Europe: Austria, Belgium, France, Germany, the Netherlands and Switzerland. Southern Europe: Italy, Portugal, Serbia, Slovenia, Spain. Eastern Europe: Bulgaria, Czech Republic, Hungary, Poland, Republic of Moldavia, Romania, Russian Federation, and Ukraine. Africa: Egypt and Réunion. Asia: China, Indonesia, Israel, Japan, India, Islamic Republic of Iran, Pakistan, Republic of Korea and Turkey. Oceania: Australia and New Zealand. North America: Canada and the United States. South America: Brazil and Chile [23]. **c** SMA type. **d** Age group

The CTSR retrieved data from 221 sites in 42 countries holding information on 6559 clinically diagnosed patients. The results by region, SMA type (I–III) and age group are summarized in Fig. 2. The CTSR does not collect gender data.

Comparing the patient population from both registries, similar patterns were observed. By far, the majority of patients resided in Europe (Global Patient Registry: 66%, *n* = 2976; CTSR: 59%, *n* = 3841). Almost half (Global Patient Registry: 45%, *n* = 2035; CTSR: 48%, *n* = 3130) of patients were diagnosed with SMA type II, whereas less than 20% (Global Patient Registry 18%, *n* = 833; CTSR: 16%, *n* = 1028) were classified as type I. This is also partly reflected in the age distribution of the patients. Infants and toddlers (aged 0–2 years), the age group to which most SMA type I patients belong, comprised only ~13% (Global Patient Registry: 12%, *n* = 544; CTSR: 14%, *n* = 898) of all patients in these registries. The majority of patients (Global Patient Registry: 39%, *n* = 1783; CTSR 36%, *n* = 2389) were children (3–11 years), followed by adults of 18–45 years of age (Global Patient Registry 26%, *n* = 1161; CTSR: 26%, *n* = 1682) and adolescents (12–17 years; Global Patient Registry: 13%, *n* = 611; CTSR: 21%, *n* = 1357). The gender distribution of patients in the Global SMA Patient Registry was nearly equal.

The ratio of prevalent SMA cases who are easily approachable to the population was calculated from the retrieved patient and population numbers in each country (Table e-1). There was considerable inter-country variability in this prevalence, ranging from 0.01 to 2.43 per 100,000 (Global Patient Registry), respectively, 0.00 to 4.11 per 100,000 (CTSR).

## Discussion

Spinal muscular atrophy is one of the leading genetic causes of infant mortality and represents a significant healthcare burden. With the development of promising new therapies for this condition [24, 25], comes the need for an improved understanding of its epidemiology and the access to specialized care. TREAT-NMD is a global network that plays a key role in addressing these important issues. To date, no global epidemiological studies of genetically confirmed SMA have been performed. Information is scarce and derives mainly from a limited number of regional studies, often predating genetic testing results, or from estimations based on carrier frequencies obtained from larger population cohorts.

In the absence of large-scale surveillance for SMA, which appears not feasible at present, a novel, international, multi-source approach was used. This approach enabled us to estimate the SMA incidence in multiple countries and to gain insight regarding the portion of the SMA population which is able and willing to participate in SMA research.

Whilst we initially contacted laboratories across the globe, response rates in European countries exceeded other parts of the world. There are several potential reasons for the lack of response or data collection from other countries. Whilst a reliable database (Orphanet) listing the majority of laboratories in European countries exists, for other continents, this is not the case, and our identification of genetic laboratories from non-European countries may, therefore, not have been as robust. Second, publicly owned laboratories, which are common in Europe, more often provided data than those which were privately owned. Furthermore, the response rate was highly improved by contacting laboratories via native speakers and local contacts, which was supported by the infrastructure provided by TREAT-NMD. This observed variability in response rates highlights the importance of multicentre, multinational collaboration in the rare disease field.

Estimated incidence of genetically confirmed SMA patients in 18 European countries ranged from 1 in 3900 to 16,000. There are two sources of data to compare our findings with: studies of genetically confirmed cases observed in the clinic and studies of carrier rates, which in some cases provide projections of potential cases in the population. Incidence estimates based on carrier screening yield higher estimates than population-based studies of observed cases. Wilson and Ogino projected an incidence of 1 per 6000 live births (~16.7 per 100,000 live births) from a carrier frequency estimate and a summary incidence of 9.7–10.1 per 100,000 live births, estimated from 15 studies of clinically diagnosed cases observed between 1960 and 1996 [15, 18]. Similarly, Jedrzejowska et al. observed in Poland a birth incidence of 1 per 9749 births (10.3 per 100,000 live births) but projects an incidence of 1 per 4900 live births (20.4 per 100,000 live births) from carrier frequencies [14]. There are several reasons that could cause differences between population-based incidence and incidence projected from carrier frequencies. The latter could be an underestimation because of de novo mutations (~2% of SMA patients [26]), limitations of diagnostic testing that cannot detect point mutations (~5% of all mutations [2]), and multiple copies of *SMN1* on the same chromosome [27], resulting in higher false-negative rates if only *SMN1* copy numbers are counted [28]. However, it can also be an overestimation due to greater genetic testing among persons with a higher risk of SMA, a high rate of foetal death due to the disease severity, and lethality of the absence of *SMN1* and its homologue *SMN2*, absent in 10–15% of the general population [16]. Furthermore, there are reports of unaffected individuals with no functional *SMN1* copies [29–31]. High rates of consanguineous marriages in some countries/communities may contribute to the variation in estimations.

Variability between countries included lower incidences in some Northern and Western European countries and higher incidences in other European countries. It is important to note that the responses to the questionnaire indicate the laboratory location and not necessarily the residency of the patient. Some of the variability in incidence rates may, therefore, be accounted for by cross-border testing by the laboratories. In Germany, several laboratories have indicated that they perform cross-border testing, which could account for the higher reported incidence there (26.7 per 100,000). This could also be the case for the relatively higher rate observed in Croatia, where some neighbouring countries do not provide laboratory testing for SMA. Conversely, some laboratories in the countries which show relatively lower incidence rates, e.g., the UK and The Netherlands, also test samples from abroad. In addition, despite only testing nationally, we found a relatively higher incidence in France, Italy, Bulgaria, and Hungary. Therefore, our incidence variability cannot be explained by cross-border testing alone. There are a few other limitations to our method of incidence estimation. Only countries with a high response rate were used when calculating the incidence. Furthermore, not all laboratories in those countries responded to the survey; however, for the countries that we took into account, local experts’ advice was utilised to ensure that the study included the main laboratories, e.g., in Italy, it is not certain that all of the remaining laboratories do test for SMA, and if they do, it will only concern a small number of tests. Second, some calculations are based on a low number of patients and live births, such as in countries with relatively small populations like Bulgaria and Hungary. In those cases, small changes in population and the number of diagnosed patients per year will have a relatively large effect on the incidence rate. Third, many laboratories cannot test for point mutations, which means that those patients might not be included in the calculations. However, some laboratories indicated that these samples were sent elsewhere for further testing and point mutations have a very low occurrence [2]. Other contributing factors for regional variation may include differences in genetic testing availability and screening practices, genetic confirmation of prevalent cases that previously only had clinical diagnosis, gene pools or rates of consanguinity, changes in the population composition, or clinical trial screening. Incidence was especially low in Greek-Cyprus and Ireland. Both countries are very small. In Cyprus, the level of genetic testing is relatively low and there is a high level of misdiagnosis. In case of the Irish SMA patients, it is possible that some of them are diagnosed in the United Kingdom. However, as the Irish population is relatively small, the number of the additional patients that might have been diagnoses in the United Kingdom would not have great impact on the results from the United Kingdom.

To estimate the size of the readily approachable and research ready SMA population, we conducted enquiries into the TREAT-NMD Global Patient Registry and the CSTR. Not unexpectedly, our findings show a subset of the SMA population prevalence found in the literature, approximately 2–5 times less [13, 19]. First, available literature mostly predates genetic testing, whereas the patients we included from registries were only those with genetic confirmation. In addition, some registries have only recently been established, and are expected to contain more patients in the future. The majority of participating registries have been set up with clinical trial recruitment in mind; therefore, patients not interested in trial participation may decide not to sign up. All registries provide data to TREAT-NMD voluntarily and lack of response of some registries may be due to a lack of resources. Furthermore, in the CTSR only specialist medical centres voluntarily enter data and not all SMA patients attend those centres.

Type I SMA, the most severe clinical presentation, is the most common subtype [15]. However, in both the patient registry and the CTSR, less than 20% of cases were classified as type I. Type I patients have a short life expectancy (<2 years of age), which will not only decrease their chance of being alive on the prevalence day, but may also reduce the likelihood of being registered by their parents in the patient registry.

We observed significant variability in the numbers of registered patients. Possible reasons for this include the healthcare infrastructure in the country affecting access to genetic testing and care, the year of setup, budget, number of staff and purpose of the registry (e.g., regulatory requirements or research/autonomous initiatives), and who is responsible for data entry (patients/guardians or professionals). No clear relationship was observed between our findings and any one of these variables (data not shown); it is likely that the variability is due to a combination of factors.

Data derived from the genetic laboratories and the registries represent unique data sets and cannot easily be compared. It is difficult to convert incidence into prevalence unless life expectancy is known, and given that SMA is a heterogeneous disease [8] with differences in standards of care between countries, it is difficult to calculate a clinically meaningful average life expectancy. The calculations are also based on small patient populations. Nonetheless, the number of patients diagnosed genetically is generally comparable or even higher than reported in previous epidemiological studies [13–17], at least in countries, where genetic testing is readily available.

SMA is a complex neurodegenerative disease which requires a comprehensive, multidisciplinary approach to ensure the best medical care and clinical outcomes [32]. Our findings from the Global Patient Registry and the CTSR indicate that many patients are not registered at specialized neuromuscular centres, and do not self-identify via patient registries, and thus may not have access to research study opportunities, the best standards of care and advanced treatment.

With a growing number of therapies being developed, there is an increasing need for reliable and larger scale SMA incidence estimations. We provide a novel method of estimating SMA incidence utilizing multiple sources. As this is the first time, a higher incidence has been studied and reported in these countries, these findings require replication with a population-based study. This study is a step forward in understanding the epidemiology of SMA and number of patients that are ready to participate in trials for new, innovative therapies or observational research and presents potentially new hypotheses to test with regard to the countries where we identified a higher than anticipated incidence.

## Electronic supplementary material

Below is the link to the electronic supplementary material.

Supplementary material 1 (DOC 101 kb)
