# Supplementary material for: A multi-source approach to determine SMA incidence and research ready population
Source: J Neurol. 2017 Jun 20;264(7):1465–73. doi: 10.1007/s00415-017-8549-1 (PMC5502065; doi:10.1007/s00415-017-8549-1)
Supplement: Supplementary file 1 — Supplementary material 1 (DOC 101 kb) [file 415_2017_8549_MOESM1_ESM.doc]

**Supplementary data**

**Table e-1** Prevalence of the research ready population from the Global SMA Patient Registry and the CTSR

|  |  | **Registry** | | **CTSR** | |
| --- | --- | --- | --- | --- | --- |
| **Country** | **Population** | **No of patients** | **Prevalence (per 10^5^)** | **No of patients** | **Prevalence (per 10^5^)** |
| Finland | 5,503,457 | 14 | 0.25 | 35 | 0.64 |
| Sweden | 9,779,426 | - | - | 61 | 0.62 |
| Norway | 5,210,967 | 15 | 0.29 | 25 | 0.48 |
| Denmark | 5,669,081 | 138 | 2.43 | 233 | 4.11 |
| United Kingdom | 64715810 | 408 | 0.63 | 614 | 0.95 |
| Ireland | 4,688,465 | 20 | 0.43 | 0 | 0.00 |
| the Netherlands | 16,924,929 | 208 | 1.23 | 137 | 0.81 |
| Belgium | 11,299,192 | - | - | 176 | 1.56 |
| France | 64,395,345 | - | - | 196 | 0.30 |
| Germany | 80,688,545 | 346 | 0.43 | 701 | 0.87 |
| Austria | 8,544,586 | 34 | 0.40 | 26 | 0.30 |
| Switzerland | 8,298,663 | 72 | 0.87 | 102 | 1.23 |
| Portugal | 10,349,803 | - | - | 26 | 0.25 |
| Spain | 46,121,699 | 138 | 0.30 | 106 | 0.23 |
| Italy | 59,797,685 | 439 | 0.73 | 557 | 0.93 |
| Slovenia | 2,067,526 | - | - | 34 | 1.64 |
| Serbia | 8,850,975 | 107 | 1.21 | 46 | 0.52 |
| Bulgaria | 7,149,787 | 59 | 0.83 | 46 | 0.64 |
| Romania | 19,511,324 | - | - | 26 | 0.13 |
| Hungary | 9,855,023 | 85^a^ | 0.86 | 82 | 0.83 |
| Slovakia | 5,426,258 | 13 | 0.24 | - | - |
| Czech Republic | 10,543,186 | 86 | 0.82 | 123 | 1.17 |
| Poland | 38,611,794 | 339 | 0.88 | 191 | 0.49 |
| Moldova, Republic of | 4,068,897 | - | - | 21 | 0.52 |
| Ukraine | 44,823,765 | 167 | 0.37 | 145 | 0.32 |
| Lithuania | 2,878,405 | 9 | 0.31 | - | - |
| Russian federation | 143,456,918 | 279 | 0.19 | 132 | 0.09 |
| Réunion | 861,154 | - | - | 9 | 1.05 |
| Turkey | 78,665,830 | 265 | 0.34 | 448 | 0.57 |
| Israel | 8,064,036 | - | - | 21 | 0.26 |
| Egypt | 91,508,084 | - | - | 31 | 0.03 |
| Iran, Islamic Republic of | 79,109,272 | - | - | 54 | 0.07 |
| Pakistan | 188,924,874 | - | - | 2 | 0.00 |
| India | 1,311,050,527 | - | - | 48 | 0.00 |
| Indonesia | 257,563,815 | - | - | 1 | 0.00 |
| China | 1,376,048,943 | 179 | 0.01 | 338 | 0.02 |
| Korea, Republic of | 50,293,439 | - | - | 1 | 0.00 |
| Japan | 126,573,481 | - | - | 94 | 0.07 |
| Australia | 23,968,973 | 16 | 0.07 | 69 | 0.29 |
| New Zealand | 4,528,526 | 34 | 0.75 | 40 | 0.88 |
| Canada | 35,939,927 | 84 | 0.23 | 136 | 0.38 |
| United States | 321,773,631 | 738 | 0.23 | 1 416 | 0.44 |
| Mexico | 127,017,224 | 41 | 0.03 | - | - |
| Brazil | 207,847,528 | 60 | 0.03 | 3 | 0.00 |
| Argentina | 43,416,755 | 133 | 0.31 | - | - |
| Chile | 17,948,141 | - | - | 7 | 0.04 |

Countries ordered by geographical location.

^a^ 1^st^ September 2014 (population 9,889,540 people).
